# Regional variation in COVID-19 vaccine uptake and intention in Nigeria: A computer assisted telephone survey

**DOI:** 10.1371/journal.pgph.0002895

**Published:** 2024-11-21

**Authors:** Eniola Adetola Bamgboye, Rawlance Ndejjo, Nuole Chen, Rotimi Felix Afolabi, Steven N. Kabwama, Mobolaji M. Salawu, Segun Bello, Ayo Stephen Adebowale, Magbagbeola David Dairo, Lily L. Tsai, Rhoda K. Wanyenze, Olufunmilayo Ibitola Fawole

**Affiliations:** 1 Faculty of Public Health, Department of Epidemiology and Medical Statistics, College of Medicine, University of Ibadan, Ibadan, Nigeria; 2 Department of Disease Control and Environmental Health, School of Public Health, College of Health Sciences, Makerere University, Kampala, Uganda; 3 Department of Political Science, Massachusetts Institute of Technology, Cambridge, Massachusetts, United States of America; 4 Department of Community Health and Behavioral Sciences, School of Public Health, College of Health Sciences, Makerere University, Kampala, Uganda; New York University Grossman School of Medicine, UNITED STATES OF AMERICA

## Abstract

Globally, vaccination has been known to reduce the burden of infectious diseases such as COVID-19, with its effectiveness relying on public acceptance and widespread uptake. Despite the efforts put into the rapid development of SARS-COV-2 vaccines, uptake remains a major challenge in many countries especially those with large population dynamics like Nigeria. Most studies in Nigeria have assessed the uptake of the SARS-COV-2 vaccine among groups of people without consideration for ethno-cultural diversity. This study therefore explored COVID-19 vaccine uptake, its determinants and future intention among adult Nigerians in the six geopolitical zones of Nigeria. This was a cross-sectional survey using a *Computer Assisted Telephone Interview*. The study enrolled 1148 adults from one representative state across each of the six geopolitical zones that had a high COVID-19 burden. Trained research assistants interviewed consenting respondents using a pretested semi-structured questionnaire configured in Survey CTO between May and June 2022. Descriptive statistics were provided as means for continuous variables, while categorical variables were expressed as frequencies and percentages. Prevalence ratios were used as the measure of association. Respondents’ age was 37.8±13.4 years, 53.9% were men and majority (65.2%) from southern Nigeria. About half (50.9%) of the respondents had not received vaccination, 29.7% were fully vaccinated, and 19.3% had incomplete dose. Majority (65.5%) of the respondents in the Northern states had not received SARS-COV-2 vaccine. Participants’ age, sex, place of residence, occupation, religion, and region of residence were associated with vaccine uptake (p<0.05). About 70% of respondents who had not received the vaccine had the intention to receive the vaccine. A low rate of SARS-COV-2 vaccine uptake, particularly in the Northern region, and a high level of intention to receive the vaccine were reported among adult Nigerians. Focused efforts are needed in the Northern region to enhance SARS-COV-2 vaccine uptake.

## Background

The novel coronavirus disease caused by the SARS-COV-2 virus became a global pandemic with a disease burden that increased rapidly since December 2019, when the first human case was discovered in Wuhan China [1,2]. Globally, the World Health Organization (WHO) reported a total of 772 million confirmed cases and 6.9 million deaths as of November 19, 2023. In the WHO African region, 47 countries had been affected by the COVID-19 disease with over 6.9 million confirmed cases and 175,000 deaths [3,4]. Nigeria was among the first countries in sub-Saharan Africa to identify COVID-19 cases and had recorded a total of 266,675 confirmed cases and 3,567 deaths as of November 30, 2023 [5].

Nigeria was categorized as one of the 13 high-risk African countries with respect to the spread of COVID-19 and among the vulnerable African nations, given the weak state of the healthcare system [6]. To mitigate the spread of COVID-19 globally, various public health preventive measures and non-pharmaceutical intervention (NPIs) were implemented in line with the WHO guidelines. Some of the measures included restriction of movement such as lockdowns, promotion of respiratory hygiene (masking, coughing/sneezing etiquette) and hand hygiene practices [7].

Despite these preventive measures for COVID-19, cases continued to rise in Nigeria and other high-risk nations suggesting community transmission of the disease. One of the important preventive measures to reduce the community spread and curtail adverse effects of infectious diseases without definitive treatment such as COVID-19 is by improving the herd immunity of the community through vaccination. Vaccines are biological preparation that confers immunity or protection to individuals against certain infectious organisms. They also prevent and reduce the spread of infection by improving population level immunity known as herd immunity. This implies that when a large proportion of a population are immunized, they confer protection on the unimmunized in that population thereby breaking the chain of transmission of the infection and hindering further spread of the disease [8–10].

In response to the urgent need to control the spread of the virus, there was a need for prompt and expedited development of the SARS-COV-2 vaccine which was achieved within one year of the onset of the pandemic as against the usual development and commercialization of vaccine which takes at least 5 years [11]. As at December, 2022, over 60 vaccines underwent clinical trial with about 34 available for public use- including Pfizer BioNTech, Moderna, Johnson and Johnson, Astrazeneca, Janssen, Sinopharm, BIBP, Sputnik V, CoronaVac, Novavax, Covaxin etc [12]. As of 4 December 2022, a total of 291 million people in the African Region had completed the primary COVID-19 vaccination series, representing 24.9% of the Region’s population [3].

On March 2, 2021, the Federal Government of Nigeria received 3.92 million doses of the Oxford/AstraZeneca vaccine, shipped via the COVAX Facility, which was a partnership between Coalition for Epidemic Preparedness Innovations (CEPI), GAVI, UNICEF and WHO [13]. The first and second phases of vaccination commenced on March 5, and August 17, 2021 respectively [13,14]. The Federal government of Nigeria started by vaccinating frontline healthcare workers, the highest-priority recipients, followed by strategic leaders and then the general population [15]. However, the intervention was met with hesitancy and resistance by the general Nigerian populace similar to many countries around the world. The accelerated development of the vaccine created a lack of trust in the world health governing body, as well as national governments, as to the effectiveness and safety of the COVID-19 vaccine [10,16,17]. There was also heightened public anxieties as regards to possible side effects of the vaccine which are not yet known to scientists or experienced by anyone [17,18]. Unfortunately, to worsen the hesitancy to vaccine uptake in Nigeria, there had been antecedent history of mistrust in vaccination by citizens. One of the notable occurrences was the rumour surrounding the content of polio vaccines, which led to boycott of routine immunization activities in some parts of the country nearly two decades ago [19,20].

Nigeria is a multi-ethnic, multi-cultural and multi-religious country with profound differences in the regions of the country [21]. Routine vaccination coverage rates differ across the regions and states of the country including urban-rural differentials [22] These disparities have been adduced to different reasons including mistrust, educational level, political and religious differences [22,23]. Most studies conducted in Nigeria on SARS-COV-2 vaccine uptake and willingness have been at the community, state or among specialized group of people [24–27]. Only few studies have attempted to explore the regional differences in the uptake of the new SARS-COV-2 vaccine with already questionable global acceptance [28–30]. This study was therefore conducted to assess SARS-COV-2 vaccine uptake, determinants of uptake and future intention to have the COVID-19 vaccine among adult Nigerians in the six geopolitical regions. The results will help to identify the regional barriers and inform the design of suitable interventions that can facilitate adoption and adherence to vaccination during public health emergencies in Nigeria.

## Methods

Nigeria is the most populous country in Africa. Nigeria has 36 states and a Federal Capital Territory (FCT), Abuja and is distributed into six geopolitical zones (North Central, North East, North West, South East, South-South, and South West). There are six states in each geopolitical zone of Nigeria, except for the northwest, which has seven states, and the southeast, which has five states. As of 2023, Nigeria’s estimated population was projected to be 214 million people [31].

**Fig 1:**
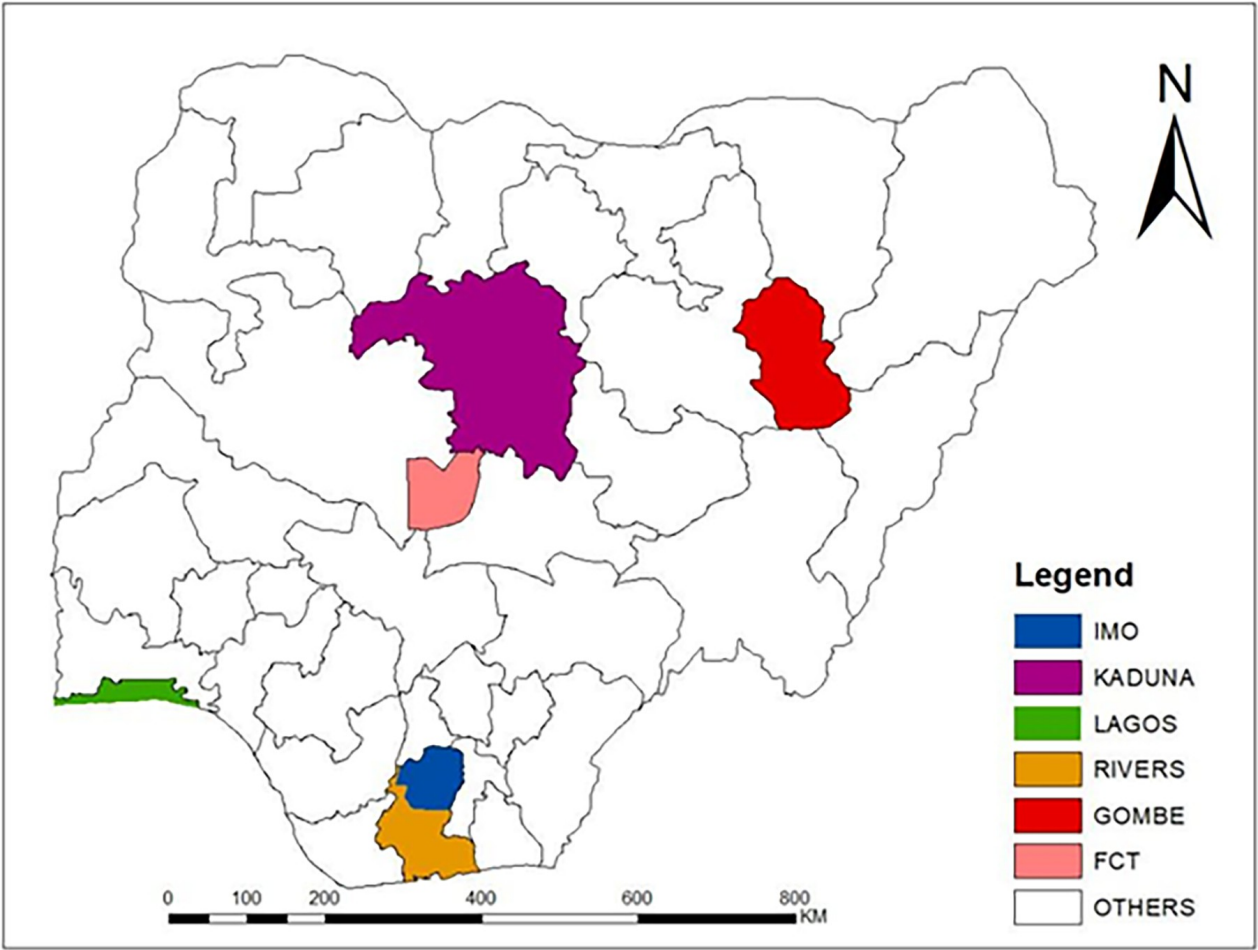
Map of Nigeria showing states selected per geopolitical region.

The first case of COVID-19 in Nigeria was identified on February 27, 2020, and by June, 27, 2022, a total of 259,377 have been recorded in all zones of the country. The state with the highest number of confirmed cases of COVID-19 in each region was purposively selected to represent the region. However, Gombe state was selected in the North-Eastern region due to reports of insecurity in other states in the zone (which may have hindered vaccination activities). Consequently, the following states with the number of confirmed COVID-19 cases at the time of survey in each zone were included in the study: Lagos (99,340) in South-West, Federal Capital Territory (28,652) in North-Central, Rivers (16,667) in South-South, Kaduna (11,258) in North-West, Gombe (3,307) in the North-East and Imo (2560) in the South-East [32] (Fig 1).

A cross-sectional survey among adult Nigerians was conducted between May and June 2022. The study enrolled persons aged 18 years and above, of both sexes sampled from the six geopolitical zones in the country. Participants were eligible for enrollment if they had lived in their current residence for at least six months while individuals who were unable to participate in the interview due to ill health were excluded from the survey.

A minimum sample size of 1048 was computed using the prevalence of willingness of COVID-19 vaccine uptake of 58.2% from a previous study among adult Nigerians [29]. In each selected state, the sample selection was based on quota sampling stratified by age, sex, and place of residence. The proportional allocation of respondents to each stratum was based on the analysis of the COVID-19 case-data from the Nigerian Centre for Disease Control (NCDC) Microsite as of May, 2022 [33]_,_ and this guided the distribution of sample to each of the stratum. Using the quotas as stratified in the microsite, a simple random sampling technique was then used to select the eligible respondents through randomization of phone contacts of individuals residing in the states. These phone contacts were obtained from a registered company’s database.

A "*Computer-Assisted Telephone Interview (CATI)*" was used to collect data using a structured questionnaire. The questionnaire was pretested among 50 randomly selected adult men and women from the database of phone numbers of residents in Ibadan, Oyo State, a State not part of the survey’s target locations. This pretesting process helped identify, correct errors and ambiguities in the questionnaire and determine the average time required for its administration. The questionnaire was initially developed in English and later translated into Nigeria’s three major languages—Hausa, Igbo, and Yoruba. The final questionnare was then programmed in SurveyCTO software in the local languages, incorporating appropriate routing and conditional logic, and uploaded onto handheld mobile tablets. Fifteen data collectors, each with at least a postgraduate qualification in health-related fields, fluency in the local language, and experience in mobile data collection, were trained to conduct the interviews. The telephone interviews lasted between 20 and 30 minutes. Data were simultaneously entered into mobile tablets as the telephone interview was conducted. Respondents who preferred to defer the time of phone interviews because they were temporarily unavailable were rescheduled after obtaining a new appointment schedule. Each day the data collectors did a preliminary check to ensure that data were successfully uploaded to the central server. To maintain quality control, 110 completed questionnaires were randomly selected, and follow-up calls were conducted by members of the research team to verify responses to specific questions in the survey. At the end of data collection exercise, the complete data were downloaded from the server and exported to Stata version 15 for analysis [34].

STATA version 15 was used for all data analyses. Descriptive statistics were provided as means for continuous variables while categorical variables were expressed as frequencies and percentages. Socio-economic status and Ministry of Health (MOH) trust index was generated as additive indices from six and seven manifest variables respectively. The socio-economic status index was based on household ownership of television, computer, sofa set, refrigerator, and cassette/CD/DVD player and access to electricity. The Ministry of Health trust index was composed of questions on whether the MOH was competent, objective, fair, consistent, sincere, faithful and well-resourced with response options on a 5-point Likert scale ranging from strongly disagree to strongly agree. The socio-economic status was divided into tertiles, while MOH trust index was dichotomized based on the blooms cut-off [35]. Vaccination uptake was defined as those who had received at least one dose of the COVID-19 vaccine, while intention to vaccinate among the unvaccinated was assessed by asking the respondents whether they would be willing to receive the SARS-COV-2 vaccine in future. To assess the factors associated with vaccination uptake and intention to vaccinate, two separate multivariable modified Poisson regressions models with robust error variance were used to determine the prevalence ratios and their corresponding 95% confidence intervals. This was done to obtain objective and unbiased inferences, as the binary outcome variables were not rare. Failing to account for the non-rarity of the binary outcome often leads to an overestimation of risk ratios when using conventional logistic regression [36]. In all the models, only variables with p-value < = 0.2 at bivariate levels were included in the final models. Prevalence ratios were the measure of association with variables with significant test conducted at 5%.

### Ethical considerations

Ethical approval was obtained from the Nigerian National Health and Research and Ethics Committee (NHREC). Confidentiality and anonymity were maintained. Verbal Informed consent was obtained from each respondent due to the method of data collection. Respondents were informed of their right to stop the interview at any point if they did not wish to continue. The telephone interviews were held in the language respondents felt most comfortable with. Data was only accessible to the investigator(s). All phones and tablets were password protected to protect respondent data and new sim cards purchased specifically for the survey were used. Data were uploaded on the server at the end of each day and at a place where interview was conducted to avoid loss of participants’ data.

## Results

### Socio-demographic characteristics of participants

A total of 1148 participants were interviewed. The mean (±SD) age of respondents was 37.8±13.4 years, and 53.9% were males. Most respondents were below 35 years (44.5%), and those aged ≥65 years (6.6%) were the least. Many respondents (47.9%) had completed tertiary school education, while 42.0% had completed secondary education. The majority of the respondents resided in the rural area (58.5%), were self-employed (51.1%), Christians (73.2%) and belonged to the medium socio-economic household (47.5%). Most respondents were recruited from Lagos state (46.3%), followed by the FCT (21.2%) and then Rivers state (13.4%) (Table 1).

**Table 1:** Sociodemographic characteristics of respondents (N = 1148).

	State						
Characteristics	Abuja n = 243	Gombe n = 58	Kaduna n = 100	Imo n = 61	Rivers n = 154	Lagos n = 532	Total (n = 1148)
**Age group(yrs)**	n (%)	n (%)	n (%)	n (%)	n (%)	n (%)	n (%)
<25	43(17.7)	13(22.4)	29(29.0)	11(18.0)	31(20.1)	68(12.8)	195(17.0)
25–34	75(30.9)	21(36.2)	27(27.0)	18(29.5)	40(26.0)	135(25.4)	316(27.5)
35–49	74(30.5)	16(27.6)	23(23.0)	21(34.4)	51(33.1)	209(39.3)	394(34.3)
50–64	36(14.8)	6(10.3)	14(14.0)	7(11.5)	24(15.6)	80(15.0)	167(14.6)
≥65	15(6.2)	2(3.5)	7(7.0)	4(6.6)	8(5.2)	40(7.5)	76(6.6)
**Sex**							
Male	145(59.7)	39(67.2)	59(59.0)	35(57.4)	95(61.7)	246(46.2)	619(53.9)
Female	98(40.3)	19(32.8)	41(41.0)	26(42.6)	59(38.3)	286(53.8)	529(46.1)
**Location**							
Urban	100(41.2)	45(77.6)	40(40.0)	23(37.7)	59(38.3)	209(39.3)	476(41.5)
Rural	143(58.9)	13(22.4)	60(60.0)	38(62.3)	95(61.7)	323(60.7)	672(58.5)
**Education**							
No formal	8(3.3)	1(1.7)	4(4.0)	0(0.0)	3(2.0)	12(2.3)	28(2.4)
Primary	16(6.6)	7(12.1)	14(14.0)	2(3.3)	7(4.6)	42(7.9)	88(7.7)
Secondary	110(45.3)	22(37.9)	37(37.0)	29(47.5)	74(48.1)	210(39.5)	482(42.0)
Tertiary	109(44.9)	28(48.3)	45(45.0)	30(49.2)	70(45.5)	268(50.4)	550(47.9)
**Occupation**							
Unemployed	39(16.1)	21(36.2)	23(23.0)	12(19.7)	39(25.3)	60(11.3)	194(16.9)
Employed	63(25.9)	12(20.7)	10(10.0)	12(19.7)	40(26.0)	150(28.2)	287(25.0)
Self employed	123(50.6)	18(31)	57(57.0)	34(55.7)	60(39.0)	294(55.3)	586(51.1)
Casual labourer	5(2.1)	1(1.7)	4(4.0)	2(3.3)	3(2.0)	16(3.0)	31(2.7)
Farmer	9(3.7)	5(8.6)	1(1.0)	0(0.0)	10(6.5)	0(0.0)	25(2.2)
Others	4(1.7)	1(1.7)	5(5.0)	1(1.6)	2(1.3)	12(2.3)	25(2.2)
**Socio Economic Status (SES)**							
Low	92(38.0)	30(51.7)	34(34.0)	23(37.7)	49(31.8)	158(29.7)	386(33.7)
Medium	109(45.0)	20(34.5)	37(37.0)	26(42.6)	82(53.3)	271(50.9)	545(47.5)
High	41(16.9)	8(13.8)	29(29.0)	12(19.7)	23(14.9)	103(19.4)	216(18.8)
**Religion**							
Christianity	189(77.8)	36(62.1)	21(21.0)	60(98.4)	148(96.1)	386(72.6)	840(73.2)
Muslim	48(19.8)	22(37.9)	79(79.0)	0(0.0)	4(2.6)	144(27.1)	297(25.9)
Others	6(2.5)	0(0.0)	0(0.0)	1(1.6)	2(1.3)	2(0.4)	11(1.0)

### Distribution of vaccine uptake

**Fig 2:**
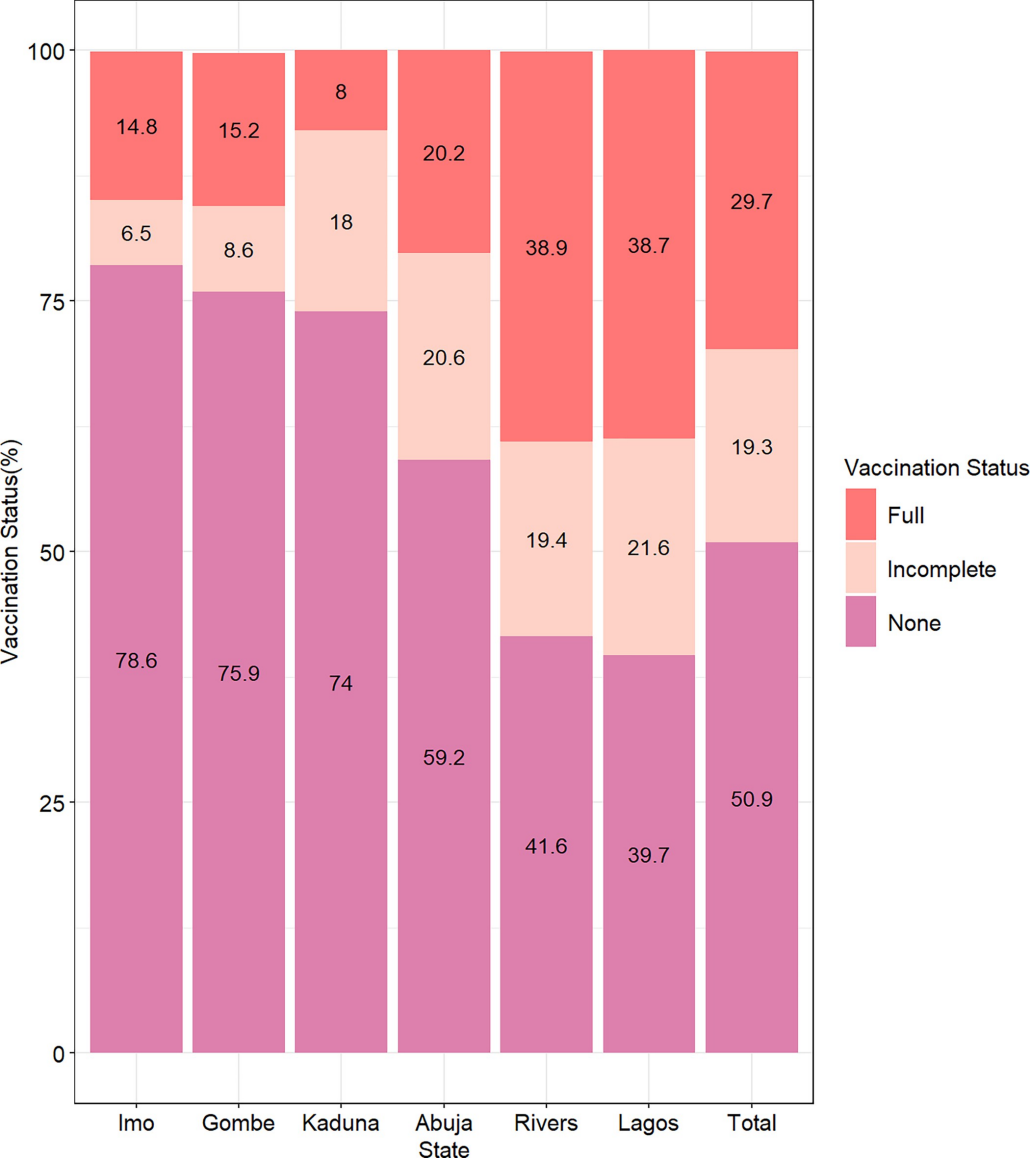
Vaccination status of respondents by States.

About one-third of the respondents (29.7%) were fully vaccinated (had received two doses), 19.3% had received an incomplete dose (received only one dose where two doses were required), while about half (50.9%) had not received the SARS-CoV-2 vaccine. Majority of the respondents from the Northern states (Gombe– 75.9%, Kaduna– 74.0%) had not received the SARS-CoV-2 vaccine with the highest non-uptake among respondents from Imo State (78.6%). A higher proportion of respondents in Rivers and Lagos had completed the dose of vaccination (Fig 2).

The highest commonly reported reason for uptake of the vaccine in all the states was to protect oneself/others from COVID-19 infection (86.9%) which was similar in all the selected states within the regions. This reason was followed by recommendation from health workers (14.2%), and high perceived risk of getting COVID-19 (9.4%). Concerning those that received vaccination due to recommendation from health workers, a higher proportion was seen in Imo State (South Eastern Nigeria) as compared to other states/regions. However, no respondent in Kaduna (North Western Nigeria) reported perception of themselves being at risk as the reason for vaccine uptake On the other hand, majority of the respondents reported they refused to take the vaccine because of “safety concerns/fear” (25.5%) which was mostly reported in Lagos (30.3%) and least reported in Kaduna (16.2%). About 24.1% of the participants reported “not having time”, while 17.3% reported “vaccines being unavailable” as other reasons for non-uptake of the vaccine. Furthermore, about a tenth (11.9%) reported “not knowing where to obtain the vaccine” as the main reason for non-uptake of the vaccine and this was found to be highest in Kaduna (24.3%) and Gombe (20.5%) states located in Northern Nigeria (S1 Table).

### Factors associated with vaccine uptake by region/state

Table 2 shows the distribution of vaccine uptake by the selected socio-demographic characteristics. More than half of the participants in the age group 35–49 years (54.1%), 50–64 years (59.9%) and >64 years (59.2%) respectively had received SARS-CoV-2 vaccine compared to those less than 35 years. More female participants (53.9%) and respondents who resided in urban areas (55.3%) had received SARS-CoV-2 vaccine. Participants’ age, sex, place of residence, occupation, religion, and residence were associated with vaccine uptake status (p<0.05). The proportion of participants who had received SARS-CoV-2 vaccine was negatively associated with education, where vaccine uptake of 68.0% and 47.6% was reported by respondents who had no formal and higher education respectively (p>0.05). Among the employed, 54.7% reported that they had received the vaccine which was slightly lower than 64.0% of farmers who reported receiving the COVID-19 vaccine (p <0.05). In addition, Imo State (South Eastern region) had the highest number of respondents with non-uptake of the SARS-CoV-2 vaccine in the southern region, while all the other northern states had a significantly higher number of respondents who had not received the vaccine as compared to south-south and south west. (p<0.001). Concerning trust in the MOH, about six out of ten respondents had a high trust (63.4%) and a statistically higher proportion (56.65%) of them had received the vaccine as compared to 37.4% of those with low trust in ministry of health (p<0.05). Also in Table 2, the unadjusted and adjusted Prevalence Ratios (aPR) of vaccine uptake are presented using the modified Poisson regression model with robust variance. Being a female (PR = 1.20; CI: 1.07,1.35), employed (PR = 1.22; CI: 1.01,1.47), and attaining a post primary education level was associated with receiving the SARS-COV-2 vaccine. After controlling for the other variables, results revealed that age, location, religion and state of residence significantly influenced SARS-CoV-2 vaccine uptake. Specifically, the likelihood of being vaccinated increased by age; participants aged 50–64 years (aPR = 1.41; CI: 1.12,1.78) had a 41% higher proportion of vaccinated individuals compared to those aged <25 years. Rural residents (aPR = 0.81; CI: 0.72,0.91) were about 20% less likely to receive the COVID-19 vaccine compared to their urban counterparts while Muslims (aPR = 1.24; CI: 1.09,1.42) had a higher likelihood of receiving the SARS-COV-2 vaccine compared with Christians.

**Table 2:** Vaccine uptake status and factors associated by selected characteristics.

Characteristics	Vaccine Uptake Status		Unadjusted model	Adjusted model
	None (n = 585)	Received (n = 563)	PR (95% CI)	aPR (95% CI)
**Age group(yrs)**	n(%)	n(%)		
<25 (ref)	123(63.1)	72(36.9)	1	1
25–34	183(57.9)	133(42.1)	1.14(0.91,1.43)	1.13(0.90,1.42)
35–49	181(45.9)	213(54.1)	**1.46(1.19,1.80)**	**1.33(1.08,1.64)**
50–64	67(40.1)	100(59.9)	**1.62(1.30,2.02)**	**1.41(1.12,1.78)**
≥65	31(40.8)	45(59.2)	**1.60(1.23,2.08)**	**1.32(1.01,1.73)**
**Sex**				
Male	341(55.1)	278(44.9)	1	1
Female	244(46.1)	285(53.9)	**1.20(1.07,1.35)**	1.09(0.97,1.23)
**Location**				
Urban	213(44.8)	263(55.3)	1	1
Rural	372(55.4)	300(44.6)	**0.81(0.72,0.91)**	**0.81(0.72,0.91)**
**Education**				
No formal	9(32.1)	19(67.9)	1	1
Primary	39(44.3)	49(55.7)	0.82(0.60,1.13)	0.96(0.60,1.55)
Secondary	249(51.7)	233(48.3)	**0.71(0.54,0.93)**	1.05(0.67,1.64)
Tertiary	288(52.4)	262(47.6)	**0.70(0.54,0.92)**	1.09(0.70,1.72)
**Occupation**				
Unemployed	107(55.2)	87(44.9)	1	1
Employed	130(45.3)	157(54.7)	**1.22(1.01,1.47)**	0.97(0.80,1.18)
Self employed	313(53.4)	273(46.6)	1.04(0.87,1.24)	0.85(0.71,1.01)
Casual labourer	17(54.8)	14(45.2)	1.01(0.66,1.53)	0.88(0.56,1.37)
Farmer	18(36.0)	32(64.0)	**1.43(1.10,1.85)**	1.21(0.88,1.67)
**Socio Economic Status**				
Low	196(50.8)	190(49.2)	1	
Medium	266(48.8)	279(51.2)	1.04(0.91,1.18)	
High	123(56.9)	93(43.1)	0.88(0.73,1.05)	
**Religion**				
Christianity	447(53.2)	393(46.8)	1	1
Islam	132(44.4)	165(55.6)	**1.19(1.05,1.35)**	**1.24(1.09,1.42)**
**State**				
Abuja	144(59.3)	99(40.7)	1	1
Gombe	44(75.9)	14(24.1)	**0.59(0.37,0.96)**	**0.51(0.31,0.82)**
Kaduna	74(74.0)	26(26.0)	**0.64(0.44,0.92)**	0.75(0.51,1.09)
Imo	48(78.7)	13(21.3)	**0.52(0.32,0.87)**	0.67(0.40,1.11)
Rivers	64(41.6)	90(58.4)	**1.43(1.17,1.76)**	**1.37(1.09,1.72)**
Lagos	211(39.7)	321(60.3)	**1.48(1.25,1.75)**	**1.46(1.21,1.77)**
**Trust in MOH+**				
Low	243(62.6)	145(37.4)	**1**	**1**
High	292(43.4)	380(56.6)	**1.51(1.31,1.75)**	**1.34(1.15,1.56)**

Bold figures–significant at p<0.05; n–number of participants; +—missing not reported; (a)PR–(adjusted) Prevalence Ratio; CI– 95% confidence interval for (a)PR.

### COVID-19 vaccine uptake intention

**Fig 3:**
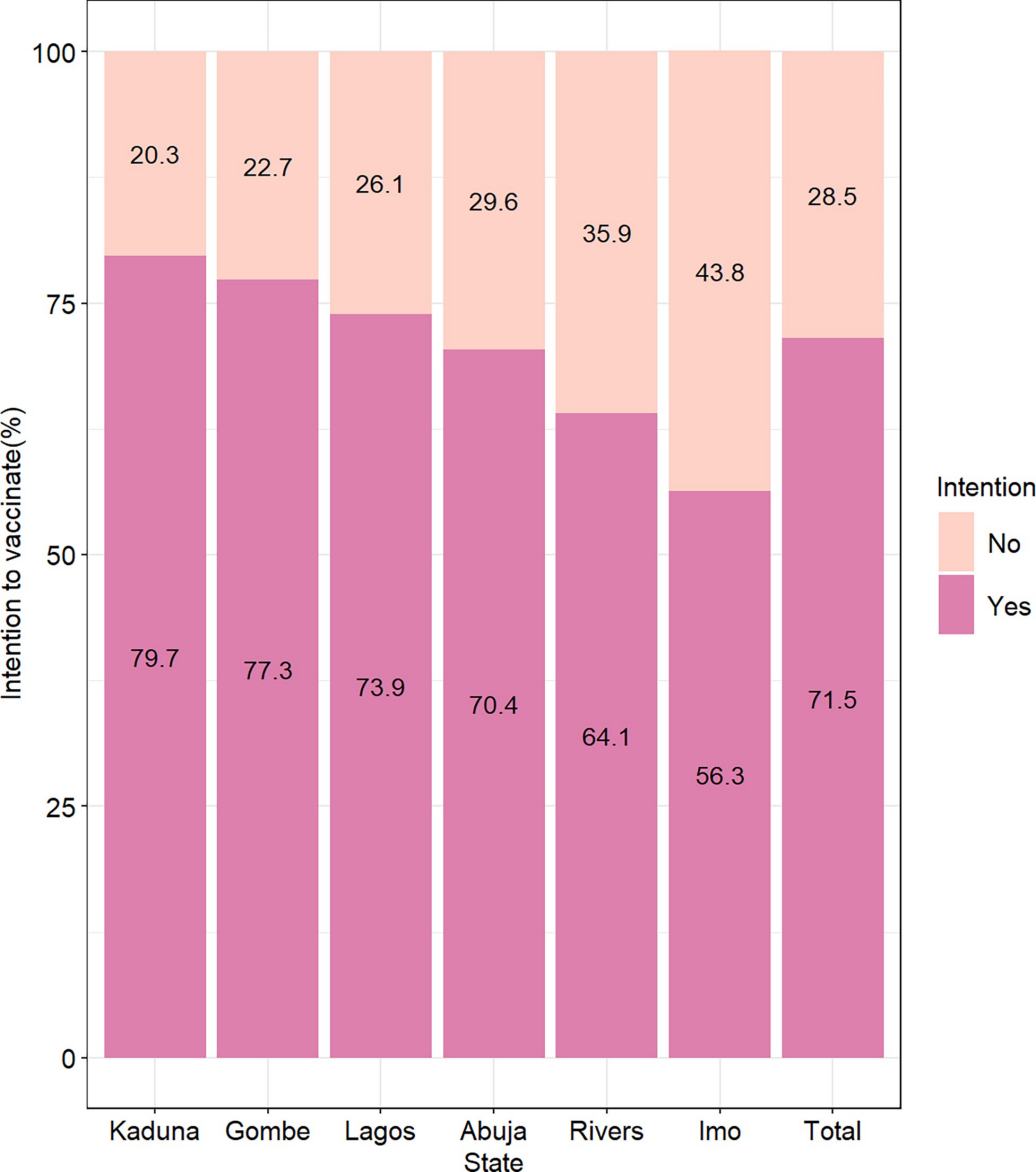
Intention of respondents to receive SARS-COV-2 vaccine by State.

Of 585 participants who were yet to take the SARS-COV-2 vaccine, 417 expressed intentions to receive the vaccine (71.5%). The proportion of those who intended to receive the vaccine was above 70% in all the states, except for Imo (56.3%) and Rivers (64.1%) States where less than two-thirds expressed intention to be vaccinated (Fig 3).

The most reported reason for intention to receive SARS-COV-2 vaccine was, “to protect self/others from COVID-19” 345(82.7%). Most respondents who reported this reason were seen in Gombe (97.1%) and Kaduna (93.2%) and the least in River State (68.3%). The other major reason for the intention to uptake the vaccine was for “travel purposes” 49(11.8%), which was reportedly higher in Rivers (17.1%) and Lagos States (12.8%) and least in Imo State (7.4%). Other reasons were the recommendation from Health Care workers 31(7.4%) and high perceived risk of contracting COVID-19(5.8%) (S2 Table).

Of 168 participants who did not intend to uptake the SARS-COV-2 vaccine, nearly half of the participants reported “safety concerns/fear” 76(45.8%) as the most common reason of not intending to receive vaccination, highest in Lagos (54.3%) and Kaduna (53.3.%) with the least in Abuja(35.7%), while about 20% reported “doubt in vaccine effectiveness” 33(19.9%) as the next main reason for not planning to receive the vaccine. A sizeable proportion 40(25.0%) also gave other reasons for non-vaccine uptake including belief that COVID-19 does not exist, religious beliefs, unavailability of vaccines and lack of time (S2 Table).

Table 3 presents the factors associated with intention to vaccinate using a modified poisson regression model approach. The modified Poisson regression model with robust variance showed that participants who were rural dwellers (PR = 1.17; CI: 1.04,1.31), of muslim faith (PR = 1.21; CI: 1.10,1.34), had trust in MOH (PR = 1.18; CI: 1.06,1.32) increased the prevalence of intention to vaccinate. But those who earned a monthly income above $240 USD (PR = 0.80; CI: 0.65,1.00) lowered the proportion of intention to uptake SARS-COV-2 vaccine. Controlling for the significant variables showed that education attainment was significantly associated with SARS-COV-2 vaccine uptake intention. The higher the education attainment, the lower the proportion of intention to vaccinate; participants who had attained tertiary/higher level of education (aPR = 0.76; CI: 0.60,0.96) had a 24% lower proportion of persons intending to vaccinate as compared to those who had no formal education (Table 3).

**Table 3:** Intention to vaccinate and factors associated by selected socio-demographic characteristics.

Characteristics	Intention to vaccinate		Unadjusted model		Adjusted model	
	Does not intend 166(28.5)	Intend 417(71.5)	PR (95% CI)	p-value	aPR (95% CI)	p-value
**Age group(yrs)**						
<25	30(24.4)	93(75.6)	1			
25–34	55(30.4)	126(69.6)	0.92(0.80,1.06)	0.245		
35–49	54(29.8)	127(70.2)	0.93(0.81,1.07)	0.290		
50–64	16(23.9)	51(76.1)	1.01(0.85,1.19)	0.937		
≥65	11(35.5)	20(64.5)	0.85(0.64,1.13)	0.267		
**Sex**						
Male	93(27.3)	248(72.7)	1			
Female	73(30.2)	169(69.8)	0.96(0.86,1.07)	0.450		
**Location**						
Urban	75(35.4)	137(64.6)	1		1	
Rural	91(24.5)	280(75.5)	**1.17(1.04,1.31)**	**0.008**	1.13(0.98,1.29)	0.089
**Education**						
No formal	3(33.3)	6(66.7)	1		1	
Primary	5(13.2)	33(86.8)	1.30(0.81,2.10)	0.279	0.97(0.73,1.29)	0.843
Secondary	60(24.1)	189(75.9)	1.14(0.71,1.82)	0.587	0.91(0.72,1.15)	0.420
Tertiary/Higher	98(34.2)	189(65.9)	0.99(0.62,1.58)	0.959	**0.76(0.60,0.96)**	**0.023**
**Occupation**						
Unemployed	30(28.6)	75(71.4)	1			
Employed	38(29.2)	92(70.8)	0.99(0.84,1.17)	0.912		
Self employed	88(28.1)	225(71.9)	1.01(0.88,1.16)	0.929		
Casual labourer	6(35.3)	11(64.7)	0.91(0.62,1.31)	0.602		
Farmer	4(22.2)	14(77.8)	1.09(0.83,1.43)	0.544		
**Monthly Income ($)+**						
≤72	48(24.4)	149(75.6)	1		1	
73–120	41(27.3)	109(72.7)	0.96(0.85,1.09)	0.534	0.99(0.86,1.13)	0.839
121–240	28(28.3)	71(71.7)	0.95(0.82,1.10)	0.478	1.03(0.89,1.20)	0.671
>240	24(39.3)	37(60.7)	**0.80(0.65,1.00)**	**0.047**	0.94(0.75,1.18)	0.599
**Socio-economic status**						
Low	61(31.4)	133(68.6)	1		1	
Medium	63(23.7)	203(76.3)	1.11(0.99,1.25)	0.071	1.11(0.97,1.27)	0.125
High	42(34.2)	81(65.9)	0.96(0.82,1.13)	0.620	0.99(0.81,1.20)	0.894
**Religion+**						
Christianity	140(31.4)	306(68.6)	1		1	
Islam	22(16.8)	109(83.2)	**1.21(1.10,1.34)**	**<0.001**	1.04(0.90,1.21)	0.561
**State**						
Abuja	42(29.6)	100(70.4)	1		1	
Gombe	10(22.7)	34(77.3)	1.10(0.91,1.33)	0.345	1.14(0.90,1.46)	0.272
Kaduna	15(20.3)	59(79.7)	1.13(0.97,1.32)	0.121	1.12(0.88,1.43)	0.362
Imo	21(43.8)	27(56.3)	0.80(0.61,1.05)	0.105	0.83(0.62,1.12)	0.224
Rivers	23(35.9)	41(64.1)	0.91(0.74,1.12)	0.382	0.86(0.68,1.08)	0.200
Lagos	55(26.1)	156(73.9)	1.05(0.92,1.20)	0.475	1.03(0.89,1.18)	0.725
**Trust in MOH+**						
Low			1		1	
High			**1.18(1.06,1.32)**	**0.003**	1.11(0.97,1.26)	0.131

Bold figures–significant at p<0.05; n–number of participants; +—missing not reported; (a)PR–(adjusted) Prevalence Ratio; CI– 95% confidence interval for (a)PR.

## Discussion

Vaccination is a cost-effective public health strategy for preventing and controlling the community spread of communicable diseases like COVID-19. However, despite the efforts put into the rapid development of SARS-CoV-2 vaccines, and ensuring equitable access, uptake remains a major challenge in many countries especially those with large population dynamics like Nigeria. This telephone-based survey was conducted to assess the prevalence of SARS-CoV-2 vaccine uptake among adult Nigerians, its determinants and the intention for future vaccine uptake.

Our study found that less than a third of adult Nigerians were fully vaccinated, though about half of all the respondents had received at least one dose of SARS-CoV-2 vaccine. This prevalence was consistent with findings from a national household survey conducted in Nigeria, which investigated Nigerians’ perceptions of SARS-CoV-2 vaccine uptake. This demonstrates the connection between people’s perceptions and their actual practices [37]. In addition, SARS-CoV-2 vaccine uptake was reported to be higher in the southern region as compared with the Northern regions of the country. The geographical difference in our finding is in keeping with literature on routine immunization activities in the country where Northern states have a lower vaccination coverage [38]. The lower uptake of vaccines has been reoccurring in most northern states, where the inhabitants doubt the potency of vaccines and believe they are deleterious to their well-being. These assumptions are based on past experiences that have occurred in the northern part of the country. One of these is the polio saga that occurred in 2003, where the political and religious leaders of some northern states instructed their populace not to allow their children to be immunized against polio myelitis. These leaders argued that the vaccine could be contaminated with anti-fertility agents (estradiol hormone), HIV, and cancerous agents [19]. The lower uptake of SARS-CoV-2 vaccine in the Northern region is however, worrisome, as COVID-19 disease unlike other vaccine preventable diseases, is a public health emergency and is highly communicable. To achieve herd immunity and halt the transmission of diseases like COVID-19 in future pandemics, it is essential to dispel this hoax.

Worthy to note is that Lagos State had the highest uptake of the vaccine, this is unsurprising as Lagos was the epicenter of the pandemic, had a higher number of respondents and was one of the first states to commence vaccination of the populace thus exposure to vaccines was for a longer period as compared to other states.

Top among the reasons for receiving the vaccine were self-protection which was similar across all regions suggesting the belief in the existence of the SARS-CoV-2 virus and the spread of the disease. This is commendable in view of the several rumors and conspiracy theories on COVID-19 which led to misinformation of the general populace. Nevertheless, respondents in the south south region had the highest proportion who took the vaccine because they felt they were at risk while those in the north western region felt they were not at risk. This finding was, however, similar to a national study which used a similar methodology, where respondents living in the north western region had lower odds of being vaccinated [39,40]. The low uptake of vaccination in the north western region could also be linked to insecurity issues in this region which could have hampered the roll out of vaccination campaigns.

Vaccine uptake was higher among the elderly compared to the younger age adults. This is likely due to higher risk perceptions reported by the elderly population. In addition, when vaccination of the populace commenced, the elderly was given priority thereby, creating earlier awareness among this age group. Also, there were deaths reported among some notable and popular elderly individuals in the country, which could have also impacted on the health seeking behaviour of the elderly. Furthermore, female respondents had higher vaccination rate which could be due to a better health seeking behavior among women in Nigeria as documented by previous studies [18].

Vaccination uptake was also higher among urban residents possibly due to availability and access-related factors. The ease of access plays an important role in vaccination uptake; this was demonstrated in a study conducted across several African countries where structural issues such as the availability of vaccines and distance to the nearest vaccination point were frequently reported in rural areas as barriers to receiving the SARS-CoV-2 vaccine. Access may also explain why the unemployed respondents had lower vaccination rates, as they are more likely to be in the low socio-economic class and reside in rural areas, where accessibility to proper health care services is suboptimal. Accessibility may also have contributed to the higher rates of vaccination uptake among the south west (Lagos) and south south (Rivers) states as they were among the first states to commence vaccination thus may have had better infrastructure, more resources allocated to the rollout, and greater public awareness [41].

However, the south eastern region (Imo) had a much lower vaccine uptake compared to other southern states probably due to security challenges, mandatory weekly “sit at home order” by ethnic militants and proclaimed political undertone of the non- existence of COVID-19 in the south east region [42]. This could have also imparted on the lower intention to receive the SARS-CoV-2 vaccine by inhabitants of the state, highlighting how misinformation and a negative social environment can disrupt public health interventions.

In Nigeria, the COVID-19 pandemic was accompanied by widespread counterproductive rumors and conspiracy theories [29]. These were mainly circulated on the social media and internet hence was mainly among the upper socio-economic class and thus could have influenced their perception of being at risk of COVID-19 and the uptake of the SARS-CoV-2 vaccine. Some Christian religious leaders also openly denied the occurrence of COVID-19 and admonished their followers to do same [43]. These may partly explain why those in the higher socio-economic class and Christians had a lower vaccination rate in this study.

Trust in government institutions is an important precursor for adoption of healthy behaviours especially those communicated through public health messaging. Trust and truthfulness in government represents the satisfaction of people with government performance and the perceived credibility of government recommendations such as vaccine uptake [44,45]. Our findings showed that the trust in ministry of health was a key driver in determining the level of vaccine uptake across the country.

A large proportion of the respondents who had not received the vaccine expressed an intention to uptake the vaccine. This was seen to be highest in the North Western region as compared to other states. The study by Eze et al. (2021), conducted across three regions of the country, also corroborated this finding, revealing that respondents from the Hausa ethnic group and those living in the northern region were more willing to receive the COVID-19 vaccination if recommended by health workers [46]. This may be attributed to the perceived high level of trust in the Ministry of Health in the region, as found in our study, which could influence their intention to receive the vaccine in the future. This is buttressed by previous studies which have demonstrated that trust in government health agencies improve willingness of populace to adhere to government recommendations such as adopting preventive behaviors [47,48]. In addition, the intention to receive the SARS-CoV-2 vaccine has been reported by other surveys in Nigeria to be higher in the northern part of the country [31], this could be related to the exposure to social media rumors and conspiracy theories which was widespread among the more educated southern population which could have imparted on their perception towards the safety of the SARS-CoV-2 vaccine [29].

### Strengths and limitation

Our study was nationwide involving at least one state with a high burden of COVID-19 in all geopolitical zones of the country. Thus, the study was population-based and representative of the COVID-19 burden in the different regions of the nation. Despite these strengths, response bias could be one of the critical limitations of the study, because the responses were self-reported and thus liable to social desirability bias. In addition, this was a telephone survey thus our results might reflect the SARS-CoV-2 vaccine uptake of those who have active mobile phones and present in the database. Lastly, the true prevalence of SARS-CoV-2 vaccine uptake may be lower than reported in this study, as half of the study population were from the epi center of COVID-19 and the flag bearer of the vaccination roll out in the country.

## Conclusion

Low prevalence of SARS-CoV-2 vaccine uptake and high level of intention to vaccine uptake was reported among adult Nigerians. The northern region had the lowest vaccine uptake rates. Key factors influencing increased uptake of SARS-CoV-2 vaccine were older age, female gender, urban residence, employment, Muslim religion, and residence in the southwestern and south southern regions of Nigeria. High trust in ministry of health played a significant role in enhancing vaccine uptake. among adult Nigerians. Concerted efforts need to be made to enlighten the public especially in the Northern region on the safety and need to be vaccinated against COVID-19.

## Supporting information

S1 Table Reasons for SARS-CoV-2vaccine uptake and non-uptake by states. (DOCX)

S2 Table Reasons for future intention and non-intention to receive SARS-CoV-2 vaccine by states. (DOCX)

S1 Data Datafile. (DTA)
