# Supplementary material for: Regional variation in COVID-19 vaccine uptake and intention in Nigeria: A computer assisted telephone survey
Source: PLOS Glob Public Health. 2024 Nov 21;4(11):e0002895. doi: 10.1371/journal.pgph.0002895 (PMC11581306; doi:10.1371/journal.pgph.0002895)
Supplement: S1 Table — (DOCX) [file pgph.0002895.s001.docx]

**Table A: Distribution of reasons for COVID-19 Vaccine Uptake and non-uptake by respondents in the states/region**

|  | **State** | | | | | |  |
| --- | --- | --- | --- | --- | --- | --- | --- |
|  | **Abuja**  **n=99** | **Gombe**  **n=14** | **Kaduna n=26** | **Imo**  **n=13** | **Rivers**  **n=90** | **Lagos**  **n=321** | **Total**  **(n=563)** |
| **Reason for uptake** | | | | | | | |
|  | n(%) | n(%) | n(%) | n(%) | n(%) | n(%) | n(%) |
| To protect self/others from COVID-19 | 85(85.9) | 14(100.0) | 23(88.5) | 12(92.3) | 80(88.9) | 275(85.7) | 489(86.9) |
| High perceived risk of getting COVID-19 | 10(10.1) | 2(14.3) | 0(0.0) | 2(15.4) | 9(10.0) | 30(9.4) | 53(9.4) |
| Travel purposes | 4(4.0) | 0(0.0) | 3(11.5) | 0(0.0) | 7(7.8) | 29(9.0) | 43(7.6) |
| Recommendation from Health Care Worker | 12(12.1) | 1(7.1) | 1(3.9) | 4(30.8) | 15(16.7) | 47(14.6) | 80(14.2) |
| Prioritize due to occupation | 7(7.1) | 0(0.0) | 0(0.0) | 1(7.7) | 3(3.3) | 26(8.1) | 37(6.6) |
| Prioritize due to health | 4(4.0) | 0(0.0) | 2(7.7) | 1(7.7) | 6(6.7) | 16(5.0) | 29(5.2) |
|  |  |  |  |  |  |  |  |
|  | **Abuja**  **n=144** | **Gombe**  **n=44** | **Kaduna n=74** | **Imo**  **n=48** | **Rivers**  **n=64** | **Lagos**  **n=211** | **Total**  **(n=585)** |
| **Reasons for non-uptake** | | | | | | | |
|  | n(%) | n(%) | n(%) | n(%) | n(%) | n(%) | n(%) |
| Safety concerns/fear | 33(22.9) | 9(20.5) | 12(16.2) | 13(27.1) | 18(28.1) | 64(30.3) | 149(25.5) |
| Don’t have time | 21(14.6) | 12(27.3) | 19(25.7) | 8(16.7) | 10(15.6) | 71(33.7) | 141(24.1) |
| Vaccines are unavailable | 27(18.8) | 11(25.0) | 16(21.6) | 6(12.5) | 7(10.9) | 34(16.1) | 101(17.3) |
| Don’t know where to access the vaccine | 14(9.7) | 9(20.5) | 18(24.3) | 9(18.8) | 5(7.8) | 13(6.2) | 68(11.6) |
| Doubt vaccine effectiveness | 12(8.3) | 4(9.1) | 5(6.8) | 4(8.3) | 7(10.9) | 16(7.6) | 48(8.2) |
| Do not fear COVID-19 | 9(6.3) | 0(0.0) | 3(4.1) | 4(8.3) | 9(14.1) | 6(2.8) | 31(5.3) |
| COVID-19 is a hoax | 4(2.8) | 1(2.3) | 1(1.4) | 1(2.1) | 3(4.7) | 4(1.9) | 14(2.4) |
| Religious beliefs | 3(2.1) | 4(9.1) | 0(0.0) | 1(2.1) | 0(0.0) | 3(1.4) | 11(1.9) |
| Transport Cost | 4(2.8) | 0(0.0) | 0(0.0) | 2(4.2) | 1(1.6) | 0(0.0) | 7(1.2) |
| Not among eligible group | 1(0.7) | 0(0.0) | 0(0.0) | 0(0.0) | 0(0.0) | 4(1.9) | 5(0.8) |
| Cost of vaccine | 1(0.7) | 0(0.0) | 1(1.3) | 0(0.0) | 0(0.0) | 0(0.0) | 2(0.3) |
|  |  |  |  |  |  |  |  |
