# Supplementary material for: Regional variation in COVID-19 vaccine uptake and intention in Nigeria: A computer assisted telephone survey
Source: PLOS Glob Public Health. 2024 Nov 21;4(11):e0002895. doi: 10.1371/journal.pgph.0002895 (PMC11581306; doi:10.1371/journal.pgph.0002895)
Supplement: S2 Table — (DOCX) [file pgph.0002895.s002.docx]

**Supplementary Table B: Distribution of reasons for intention to receive COVID-19 Vaccine and non-intention in future by respondents in the states/region**

|  | **States** | | | | | |  |
| --- | --- | --- | --- | --- | --- | --- | --- |
|  | **Abuja**  **n=100** | **Gombe**  **n=34** | **Kaduna n=59** | **Imo**  **n=27** | **Rivers**  **n=41** | **Lagos**  **n=156** | **Total**  **(n=417)** |
| **Reason for intention to uptake** | | | | | | | |
|  | n(%) | n(%) | n(%) | n(%) | n(%) | n(%) | n(%) |
| To protect self/others from COVID-19 | 80(80.0) | 33(97.1) | 55(93.2) | 24(88.9) | 28(68.3) | 125(80.1) | 345(82.7) |
| High perceived risk of getting COVID-19 | 5(5.0) | 7(20.6) | 4(6.8) | 1(3.7) | 1(2.4) | 6(3.9) | 24(5.8) |
| Travel purposes | 11(11.0) | 3(8.8) | 6(10.2) | 2(7.4) | 7(17.1) | 20(12.8) | 49(11.8) |
| Recommendation from Health Care Worker | 7(7.0) | 3(8.8) | 2(3.4) | 2(7.4) | 3(7.3) | 14(8.9) | 31(7.4) |
| Prioritize due to occupation | 1(1.0) | 0(0.0) | 0(0.0) | 1(3.7) | 0(0.0) | 7(4.5) | 9(2.2) |
| Prioritize due to health | 6(6.0) | 1(2.9) | 0(0.0) | 1(3.7) | 5(12.2) | 7(4.5) | 20(4.8) |
|  |  |  |  |  |  |  |  |
|  | **Abuja**  **n=42** | **Gombe**  **n=10** | **Kaduna n=15** | **Imo**  **n=21** | **Rivers**  **n=23** | **Lagos**  **n=55** | **Total**  **(n=168)** |
| **Reasons for no intention to receive vaccine** | | | | | | | |
|  | n(%) | n(%) | n(%) | n(%) | n(%) | n(%) | n(%) |
| Safety concerns/fear | 15(35.7) | 4(40.0) | 8(53.3) | 10(47.6) | 9(39.1) | 30(54.5) | 76(45.7) |
| Doubt vaccine effectiveness | 8(19.1) | 5(50.0) | 4(26.7) | 5(23.8) | 2(8.7) | 9(16.4) | 33(19.9) |
| Do not fear COVID-19 | 4(9.5) | 0(0.0) | 0(0.0) | 2(9.5) | 5(21.7) | 4(7.3) | 15(9.0) |
| COVID-19 is a hoax | 1(2.4) | 0(0.0) | 2(13.3) | 1(4.7) | 2(8.7) | 2(3.6) | 8(4.8) |
| Religious beliefs | 1(2.4) | 1(10.0) | 0(0.0) | 1(4.7) | 2(8.7) | 2(3.6) | 7(4.2) |
| Don’t have time | 0(0.0) | 1(10.0) | 0(0.0) | 0(0.0) | 0(0.0) | 4(7.3) | 5(3.0) |
| Vaccines are unavailable | 1(2.4) | 1(10.0) | 1(6.6) | 0(0.0) | 0(0.0) | 1(1.8) | 4(2.4) |
| Don’t know where to access the vaccine | 1(2.4) | 0(0.0) | 0(0.0) | 0(0.0) | 0(0.0) | 0(0.0) | 1(0.6) |
| Not among eligible group | 0(0.0) | 0(0.0) | 0(0.0) | 0(0.0) | 0(0.0) | 1(1.8) | 1(0.6) |
| Transport Cost | 0(0.0) | 0(0.0) | 0(0.0) | 0(0.0) | 0(0.0) | 0(0.0) | 0(0.0) |
| Cost of vaccine | 0(0.0) | 0(0.0) | 0(0.0) | 0(0.0) | 0(0.0) | 0(0.0) | 0(0.0) |
|  |  |  |  |  |  |  |  |
